# Mediators of Filgotinib Treatment Effects in Ulcerative Colitis: Exploring Circulating Biomarkers in the Phase 2b/3 SELECTION Study

**DOI:** 10.1093/ibd/izae278

**Published:** 2024-12-04

**Authors:** Hiroshi Nakase, Silvio Danese, Walter Reinisch, Timothy Ritter, Yan Liang, Emily Wendt, Barrett G Levesque, Oh Kyu Yoon, Yuan Tian, Luting Zhuo, Emmanuel Karouzakis, Yasmina Bauer, Alessandra Oortwijn, Toshihiko Kaise, Vladislav A Malkov, Toshifumi Hibi

**Affiliations:** Department of Gastroenterology and Hepatology, Sapporo Medical University School of Medicine, Sapporo, Japan; Inflammatory Bowel Diseases Center, Humanitas Research Hospital, Milan, Italy; Department of Internal Medicine and Gastroenterology, Medical University of Vienna, Vienna, Austria; GI Alliance Research, Southlake, TX, USA; Gilead Sciences, Inc., Foster City, CA, USA; Gilead Sciences, Inc., Foster City, CA, USA; Gilead Sciences, Inc., Foster City, CA, USA; Gilead Sciences, Inc., Foster City, CA, USA; Gilead Sciences, Inc., Foster City, CA, USA; Gilead Sciences, Inc., Foster City, CA, USA; Galapagos GmbH, Basel, Switzerland; Galapagos GmbH, Basel, Switzerland; Galapagos NV, Oegstgeest, The Netherlands; Gilead Sciences K.K., Tokyo, Japan; Gilead Sciences, Inc., Foster City, CA, USA; Center for Advanced IBD Research and Treatment, Kitasato Institute Hospital, Kitasato University, Tokyo, Japan

**Keywords:** ulcerative colitis, biomarkers, filgotinib

## Abstract

**Background:**

We utilized patient samples from the large, phase 2b/3 SELECTION trial to identify circulating biomarkers of ulcerative colitis (UC) and potential early mediators of filgotinib treatment effects.

**Methods:**

Samples were collected at baseline and during the induction phase of the SELECTION trial. Evaluated biomarkers comprised serum and stool proteins (measured by enzyme-linked immunosorbent assay), whole-blood cell counts, and whole-blood RNA-seq-derived gene-expression factors identified via exploratory factor analysis. Biomarker levels were assessed by baseline disease severity (endoscopy/bleeding/stool and Mayo Clinic Score) and biologic status (naive vs experienced). Effects of filgotinib on biomarker levels, including week 4 biomarker changes that may mediate week 10 clinical improvements, were assessed.

**Results:**

The biomarker analysis set included 598 biologic-naive patients and 592 biologic-experienced patients. Systemic inflammatory biomarkers (C-reactive protein [CRP], interleukin-6 [IL-6], serum amyloid A [SAA], and platelet cell counts) had the strongest positive correlations with baseline UC disease severity. CRP, IL-6, SAA, and neutrophil activation biomarkers (including neutrophil gelatinase-associated lipocalin [NGAL], tumor necrosis factor ɑ, and oncostatin M [OSM]), as well as platelet, neutrophil, and monocyte cell counts were increased in biologic-experienced versus biologic-naive patients. Gene-expression-derived plasmablast and cell proliferation factors were positively correlated with disease severity; B cell, T-cell activation, and plasmacytoid dendritic cell factors were negatively correlated. Filgotinib reduced nearly all proinflammatory biomarkers correlated with baseline UC disease activity; reduced SAA, CRP, IL-6, NGAL, and OSM at week 4 were identified as mediators of improved week 10 clinical scores.

**Conclusions:**

Filgotinib significantly impacted circulating biomarkers related to UC pathology. Several proinflammatory and neutrophil activation biomarkers may be early mediators of filgotinib treatment effects.

**ClinicalTrials.gov identifier:**

NCT02914522

**Key Messages**

**What is already known?**

Various inflammatory cells are associated with ulcerative colitis pathobiology, and circulating biomarkers often reflect disease patterns in the colon.

**What is new here?**

Filgotinib treatment reduced most proinflammatory biomarkers that correlated with baseline ulcerative colitis disease activity, and week 4 reductions in several biomarkers were associated with week 10 clinical score improvements.

**How can this study help patient care?**

Circulating biomarkers could be used as noninvasive, early indicators of clinical response to filgotinib treatment in patients with ulcerative colitis.

## Introduction

Ulcerative colitis (UC) is a chronic active or relapsing immune-mediated disease characterized by colonic mucosal inflammation that leads to bloody stools, frequent bowel movements, urgency, and abdominal pain.^1^ While short-term treatment goals for UC involve alleviating disease symptoms, long-term treatment goals are targeted toward inducing corticosteroid-free clinical remission and mucosal healing, and improving quality of life.^2^

A diverse range of cytokine-producing inflammatory cells contribute to UC pathology.^3^ Antigen-presenting cells induce the differentiation of naive T cells into T helper (Th) cells that release pro- and anti-inflammatory cytokines. Th17, Th9, and Th2 cells are frequently found in the lamina propria, where they produce interleukin (IL)-17, IL-9, and IL-13, respectively.^4^ There is also evidence that IL-12 and IL-18 activate Th1 cells in the lamina propria to produce tumor necrosis factor (TNF)-α, interferon (IFN)-γ, and IL-6, contributing further to UC pathology.^3^ IL-8, another key mediator of inflammation in UC, induces neutrophil migration and infiltration into the intestinal tissue, resulting in the release of inducible nitric oxide synthase and matrix metalloproteinases, leading to epithelial cell damage.^5^ Importantly, circulating biomarkers often reflect disease patterns in the colon.^6–9^

Molecular targets for the treatment of UC include TNF-α, α4β7 integrin, Janus kinases (JAKs), IL-12/23 p40, and sphingosine-1 phosphate (S1P). TNF-α antagonists, anti-α4β7 integrin antibodies, JAK inhibitors, anti-IL-12/23 p40 antibodies, and S1P modulators represent a revolutionary improvement in UC treatment. Despite these advancements in therapy, efficacy in UC treatment remains limited, with refractory disease occurring in 30%–55% of patients.^10–13^ These rates of treatment resistance likely reflect an inability to appropriately match targeted therapies in UC populations, which are heterogeneous in terms of disease phenotype and predominant immune cell and cytokine involvement. Identifying circulating biomarkers that are early mediators of treatment responses is thus desirable to inform targeted therapy selection. Studies evaluating relationships between circulating biomarkers and specific targeted therapies in UC are limited. Therefore, samples from large-scale clinical trials investigating the safety and efficacy of targeted agents provide an important opportunity to acquire such knowledge.

Filgotinib, a preferential JAK1 inhibitor, is approved in the European Union, the United Kingdom, and Japan for the treatment of moderately to severely active UC and moderately to severely active rheumatoid arthritis.^14,15^ Preferential inhibition of JAK1 by filgotinib interferes with the JAK1–signal transducer and activation of transcription (STAT) pathway and associated proinflammatory cytokine signaling.^14^ In the phase 2b/3 SELECTION trial, which enrolled biologic-naive and biologic-experienced patients with moderately to severely active UC, filgotinib 200 mg was shown to be well tolerated and efficacious in inducing and maintaining clinical remission compared with placebo.^16^ In this pre-planned analysis of SELECTION trial data, we analyzed blood-derived biomarkers (serum proteins, complete blood cell counts, and biomarkers derived from whole-blood gene expression), and fecal lactoferrin and calprotectin to evaluate biological pathways that are dysregulated in patients with UC (including in biologic-naive vs biologic-experienced individuals), the effects of filgotinib treatment (and baseline corticosteroid/immunomodulator use) on these pathways, and mediators of filgotinib treatment response.

## Materials and Methods

### Study Design and Participant Selection

Details of the phase 2b/3 SELECTION trial have been reported previously.^16^ Briefly, biologic-naive and biologic-experienced patients with moderately to severely active UC were randomized (2:2:1) to receive filgotinib 200 mg, filgotinib 100 mg, or placebo once daily for 10 weeks in 2 induction studies. Participants were permitted to use concomitant oral corticosteroids (prednisone ≤ 30 mg/day or budesonide ≤ 9 mg/day), provided the dose was stable for 2 weeks before and 14 weeks after randomization throughout the induction phase, and concomitant immunomodulators (azathioprine, 6-mercaptopurine, or methotrexate), provided the dose was stable for 4 weeks before and 10 weeks after randomization.

The current preplanned analysis of the SELECTION trial used serum, stool, and whole-blood samples collected from the induction phase of the trial. The biomarker analysis set presented in the current study excluded patients if they had a record of dose escalation of a nonstudy concomitant treatment for UC during the induction phase, had insufficient data to evaluate endoscopy/bleeding/stool (EBS) frequency (EBS remission), or failed to follow the clinical study protocol.

Samples from 19 healthy volunteers were procured by Precision Bioservices, Inc. and profiled separately. An attempt was made to match the healthy volunteers to participants in the SELECTION study. The characteristics of the recruited healthy volunteers versus participants in the SELECTION study were: aged 29–55 years (70% vs 63%); women (40% vs 47%); White (65% vs 79%), Asian (25% vs 21%), Asian Indian (10% vs 0%), and nonsmoker (90% vs 84%). All healthy volunteers, and participants in the SELECTION trial, had a body mass index less than 35 mg/kg^2^ and a fecal calprotectin level below 50 μg/g.

Because a batch effect associated with profiling healthy volunteer samples at a later time cannot be fully excluded, the biomarker levels derived from healthy volunteer samples were used for exploratory purposes only (ie, no inferential statistical tests were performed).

The SELECTION protocol was reviewed and approved by an Independent Ethics Committee or Institutional Review Board at each study site before initiation.

### Biomarker Collection and Assessments

#### Protein biomarkers

Serum and whole-blood samples were collected at baseline and at weeks 4 and 10 following initiation of filgotinib or placebo. Stool samples were collected at baseline and at week 10.

Protein biomarkers were measured by enzyme-linked immunosorbent assay at several laboratories (Table S1).

Serum protein biomarkers with at least 50% of observations within the limits of quantification, and thus included in the analyses, were calprotectin, high-sensitivity C-reactive protein (CRP), glycoprotein 130, IFN-γ, IL-17A, IL-2, IL-10, IL-22, IL-23, IL-5, IL-6, IL-6R, IL-8, neutrophil gelatinase-associated lipocalin (NGAL), oncostatin M (OSM), serum amyloid A (SAA), transforming growth factor β-1 (TGF-β1), and TNF-α (Table S1). Stool protein biomarkers comprised fecal lactoferrin and calprotectin. Standard complete blood count tests comprised platelets, monocytes, neutrophils, eosinophils, lymphocytes, and basophils.

### Hallmark Pathway Analysis of RNA-seq Data

Single-sample gene set enrichment analysis (ssGSEA) was used to calculate hallmark pathway activity scores from whole-blood RNA-seq data (Supplementary Methods),^17,18^ which capture the degree to which genes in a particular gene set are coordinately up- or downregulated within a sample. Pathway gene sets were downloaded from the molecular signature database and represent specific well-defined biological processes and display consistent gene-expression data.^19^

### Exploratory Factor Analysis of RNA-seq Data

An exploratory factor analysis (EFA) using the method of maximum likelihood to estimate factor loadings was employed to uncover the underlying structure of gene-expression drivers.^20^ Using the EFA, “unobserved” factors/drivers resulting in the production of sets of correlating genes by RNA-seq can be deduced. An RNA-seq matrix of differentially expressed genes at weeks 4 and 10 following filgotinib 200 mg treatment and a false discovery rate (FDR) of less than 0.005 were inputted into the EFA. Both optimal and parallel coordinate analyses (using nFactors package in R) were used to identify the number of required factors for EFA.

Of the factors identified, those significantly affected by filgotinib treatment were selected using a linear mixed-effects model adjusted for concomitant treatment, baseline disease severity, sex, and age. The list of factors with a clear biological interpretation was further refined using the following steps: (1) application of gene-factor loadings to an internal peripheral blood mononuclear cell atlas to highlight cell types that overexpress those genes (Figure S1B); (2) evaluation of cell specificity for the top 5 genes within the highest loadings; (3) evaluation of correlations between factors and ssGSEA scores or blood cell fractions; and (4) examination of biological functions of the top loading genes.

Following refinement, 7 factors with a clear biological interpretation were included in further analyses. These included factors that reflect the fraction of B cells, effector B cells, plasmablasts, plasmacytoid dendritic cells (pDCs), and natural killer (NK) cells, as well as transcriptional changes in cell proliferation and T-cell activation.

### Statistical Comparisons and Correlations

Details regarding the statistical correlations and comparisons are described in the Supplementary Methods.

### Mediation Analysis of Serum Protein Data

To investigate the mechanism of action of filgotinib in achieving UC clinical improvement, mediation analyses were performed using the mediation package in R. The causal model evaluated whether clinical improvements following filgotinib treatment at week 10 could be driven by an indirect path based on early changes in biomarkers from baseline to week 4 (Figure S2). Biomarker values at week 4 were grouped together by EFA and then used to examine downstream associations with clinical changes at week 10. The average causal mediation effect was defined as the expected difference in the potential outcome (clinical endpoints) when the mediator (week 4 biomarker measurements) took the value that would realize under the treatment condition, as opposed to the control condition, while the treatment status itself was held constant. *P* values for the average causal mediation effect were used to determine statistical inferences of causal association.

## Results

### Participant Disposition and Sample Availability

In the SELECTION induction studies, 659 biologic-naive and 689 biologic-experienced patients were enrolled and randomly assigned to receive filgotinib 200 mg, filgotinib 100 mg, or placebo (full analysis set [FAS]). The biomarker analysis set used in the current analysis (whereby serum and fecal protein biomarker data were available) included 598 biologic-naive (90.7%) and 592 biologic-experienced patients (85.9%). Overall, the biomarker analysis set excluded 158 patients (11.7%) from the FAS. Demographics and disease characteristics were well balanced between the filgotinib 200 mg, filgotinib 100 mg, and placebo arms in both biologic-naive and biologic-experienced patients in the biomarker analysis set (Table 1).

**Table 1.** Baseline patient demographics and disease characteristics (biomarker analysis set).

Demographic or characteristic	Biologic-naive *N* = 598			Biologic-experienced *N* = 592		
	Placebo *N* = 122	Filgotinib 100 mg *N* = 245	Filgotinib 200 mg *N* = 231	Placebo *N* = 119	Filgotinib 100 mg *N* = 244	Filgotinib 200 mg *N* = 229
Age, years, mean (SD)	40.7 (12.6)	42.4 (13.4)	42.2 (13.2)	45.4 (14.4)	43.6 (14.1)	44.0 (14.2)
Female sex, *n* (%)	46 (37.7)	105 (42.9)	116 (50.2)	47 (39.5)	79 (32.4)	99 (43.2)
Race, *n* (%)						
Asian	31 (25.4)	69 (28.2)	72 (31.2)	23 (19.3)	48 (19.7)	47 (20.5)
White	87 (71.3)	170 (69.4)	157 (68.0)	84 (70.6)	176 (72.1)	165 (72.1)
Black or African American	1 (0.8)	3 (1.2)	1 (0.4)	2 (1.7)	4 (1.6)	4 (1.7)
Other	3 (2.5)	3 (1.2)	1 (0.4)	10 (8.4)	16 (6.6)	13 (5.7)
Geographic region United States, *n* (%)	15 (12.3)	32 (13.1)	14 (6.1)	16 (13.4)	44 (18.0)	29 (12.7)
Weight, kg, mean (SD)	69.1 (15.5)	70.0 (18.2)	70.5 (17.9)	72.9 (16.7)	75.2 (17.3)	73.4 (19.0)
Height, cm, mean (SD)	169.7 (9.5)	169.0 (9.7)	168.6 (9.9)	172.2 (9.5)	172.6 (8.2)	170.0 (10.0)
BMI, kg/m^2^, mean (SD)	23.9 (4.3)	24.3 (5.1)	24.7 (5.6)	24.5 (5.2)	25.1 (5.0)	25.2 (5.8)
Duration of diagnosis years, mean (SD)	6.2 (6.7)	6.7 (7.6)	7.2 (6.8)	10.2 (7.7)	9.8 (7.4)	10.0 (7.7)
Smoking status, *n* (%)						
Former	19 (15.6)	51 (20.8)	52 (22.5)	35 (29.4)	83 (34.0)	68 (29.7)
Current	5 (4.1)	7 (2.9)	14 (6.1)	5 (4.2)	16 (6.6)	8 (3.5)
Never	98 (80.3)	187 (76.3)	165 (71.4)	79 (66.4)	145 (59.4)	153 (66.8)
Concomitant corticosteroid use, *n* (%)	39 (32.0)	74 (30.2)	70 (30.3)	52 (43.7)	110 (45.1)	103 (45.0)
Concomitant immunomodulator use, *n* (%)	35 (28.7)	74 (30.2)	71 (30.7)	27 (22.7)	54 (22.1)	59 (25.8)
Endoscopic score, mean (SD)	2.6 (0.5)	2.6 (0.5)	2.5 (0.5)	2.8 (0.4)	2.8 (0.4)	2.8 (0.4)
Mayo Clinic Score, mean (SD)	8.7 (1.3)	8.7 (1.4)	8.6 (1.3)	9.3 (1.4)	9.3 (1.2)	9.2 (1.4)

Abbreviations: BMI, body mass index; SD, standard deviation.

Whole-blood RNA samples were profiled from 574 biologic-naive patients (96.0%) and 575 biologic-experienced patients (97.1%) in the biomarker analysis set and from 19 healthy volunteers. In total, 3211 samples (including 19 from the healthy volunteers) passed quality control and were used for all subsequent analyses.

Baseline biomarker levels were compared between patients with UC (*n* = 1190) and healthy volunteers (*n* = 19), between biologic-naive (*n* = 598) and biologic-experienced (*n* = 592) patients with UC, and between patients with UC taking corticosteroids (*n* = 340) or immunomodulators (*n* = 213) at baseline and propensity-score-matched patients who were not (*n* = 340 and *n* = 213, respectively).

### Correlation of Baseline Biomarkers With UC Disease Activity

**Figure 1.**
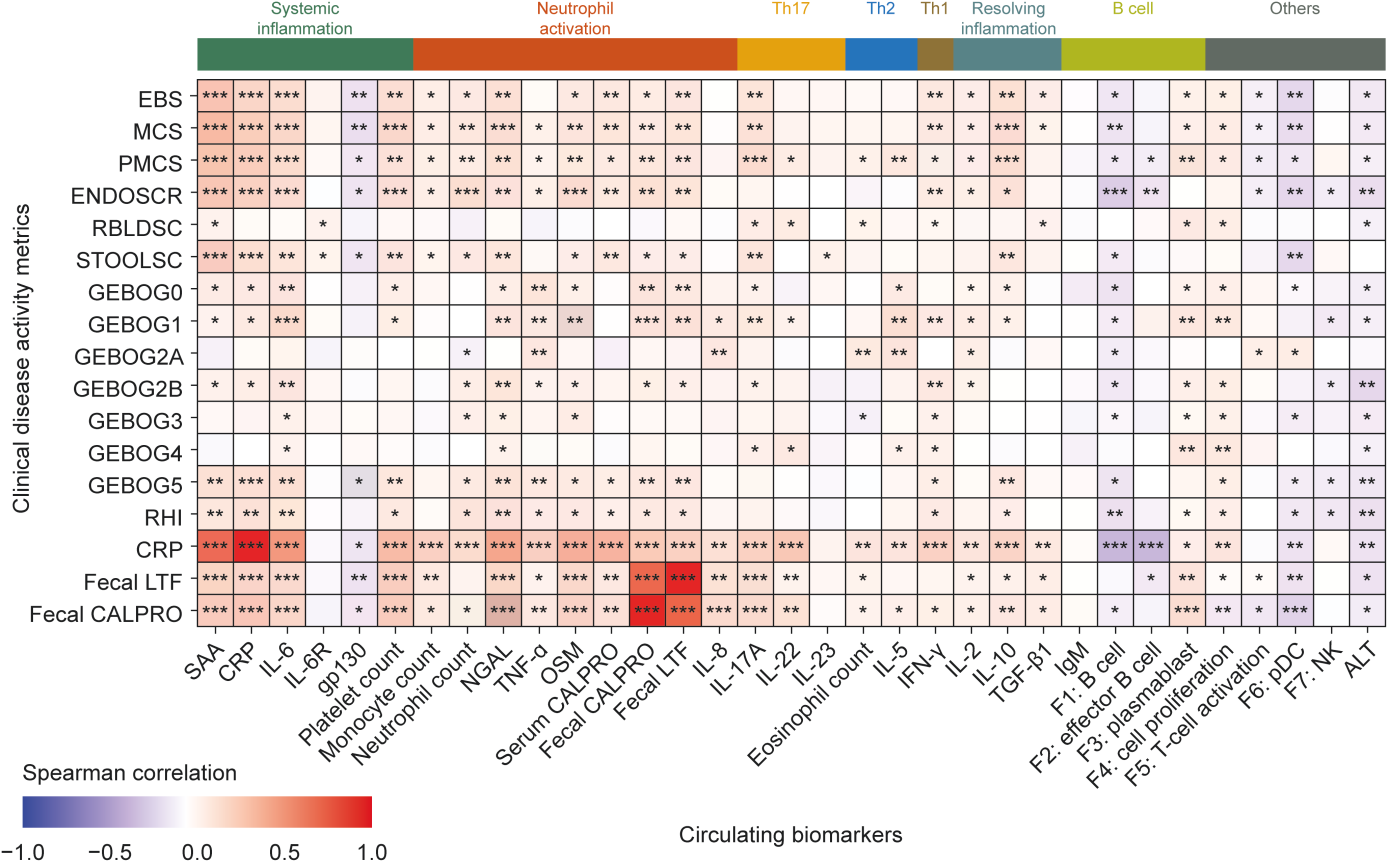
Correlation between circulating biomarkers and clinical disease activity metrics at baseline. *FDR < 1 × 10^−3^, **FDR < 1 × 10^−6^, and ***FDR < 1 × 10^−9^ as determined by Spearman’s rank correlation at baseline. Abbreviations: ALT, alanine transaminase; CALPRO, calprotectin; CRP, C-reactive protein; EBS, endoscopy/bleeding/stool; ENDOSCR, endoscopic subscore; F, factor; FDR, false discovery rate; FIL, filgotinib; GEBOG, Geboes Score components; gp, glycoprotein; IFN, interferon; Ig, immunoglobulin; IL, interleukin; IL-6R, IL-6 receptor; LTF, lactoferrin; MCS, Mayo Clinic Score; NGAL, neutrophil gelatinase-associated lipocalin; NK, natural killer; OSM, oncostatin M; pDC, plasmacytoid dendritic cell; PMCS, partial MCS; RBLDSC, rectal bleeding subscore; RHI, Robarts Histopathology Index; SAA, serum amyloid A; STOOLSC, stool frequency subscore; TGF, transforming growth factor; Th, T helper; TNF, tumor necrosis factor.

To ensure that the selected set of systemic biomarkers captured the pathobiology of UC, biomarkers were correlated with clinical disease measures at baseline (Figure 1). Systemic inflammatory biomarkers (CRP, IL-6, SAA, and platelet counts) displayed the strongest positive correlations with disease severity as assessed by EBS, Mayo Clinic Score (MCS), and endoscopic subscore. Positive correlations with disease severity (as assessed by EBS, MCS, and partial MCS) were also observed for neutrophil activation biomarkers (NGAL, OSM, serum and fecal calprotectin, fecal lactoferrin, and neutrophil and monocyte counts), Th17 (IL-17A) and Th1 (IFN-γ) cytokines, and anti-inflammatory cytokines, especially IL-10. In addition, negative correlations with most clinical disease measures as well as fecal lactoferrin and calprotectin were observed for alanine transaminase.

The lamina propria eosinophil component of the Geboes Score (GEBOG2A) was positively correlated with TNF-α levels, eosinophil counts, and IL-5 and IL-8 levels. In addition, the neutrophil component in the lamina propria (GEBOG2B) had the strongest positive correlations with IL-6, NGAL, and IFN-γ, and to a lesser extent with SAA, CRP, OSM, TNF-α, fecal calprotectin and lactoferrin, IL-17A, IL-2, and neutrophil counts.

Gene-expression-derived plasmablast and cell proliferation factors were positively correlated with disease severity as assessed by EBS, MCS, and partial MCS, whereas B cell, T-cell activation, and pDC factors were negatively correlated.

### Between-Group Comparisons of Baseline Biomarkers

#### Patients with UC versus healthy volunteers

**Figure 2.**
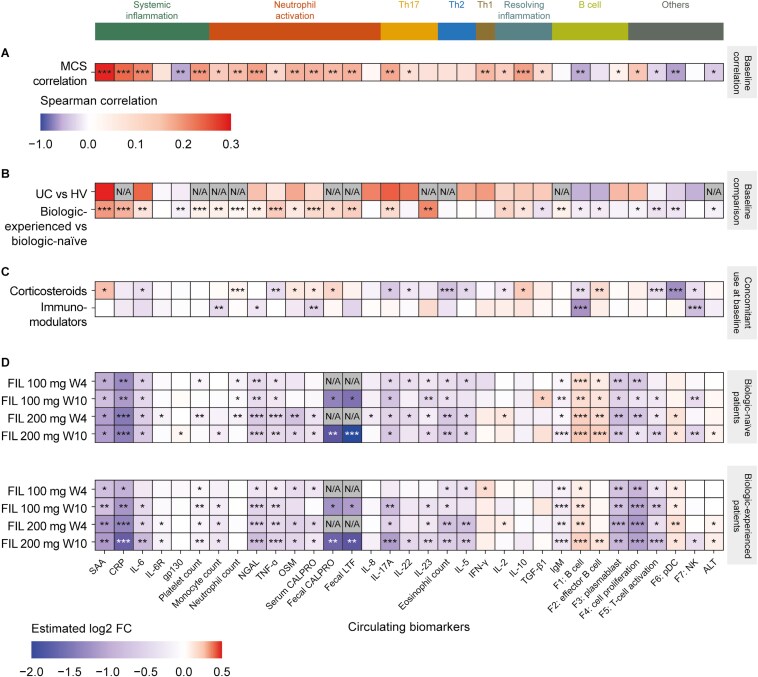
Correlation between (A) circulating biomarkers and MCS at baseline. Comparisons of circulating biomarkers at baseline in (B) patients with UC versus healthy volunteers (statistical tests for associations were not performed for these data) and patients with UC who were biologic-experienced versus biologic-naive, and (C) patients with UC taking corticosteroids/immunomodulators at baseline versus those who were not. Placebo-adjusted effects of filgotinib treatment on biomarkers at weeks 4 and 10 compared with baseline are shown in (D). *FDR < 1 × 10^−1^ **FDR < 1 × 10^−3^, and ***FDR < 1 × 10^−6^ as determined by Spearman’s rank correlation at baseline. Abbreviations: ALT, alanine transaminase; CALPRO, calprotectin; CRP, C-reactive protein; F, factor; FC, fold change; FDR, false discovery rate; FIL, filgotinib; gp, glycoprotein; HV, healthy volunteers; IFN, interferon; Ig, immunoglobulin; IL, interleukin; IL-6R, IL-6 receptor; LTF, lactoferrin; MCS, Mayo Clinic Score; N/A, not available; NGAL, neutrophil gelatinase-associated lipocalin; NK, natural killer; OSM, oncostatin M; pDC, plasmacytoid dendritic cell; SAA, serum amyloid A; TGF, transforming growth factor; Th, T helper; TNF, tumor necrosis factor; UC, ulcerative colitis; W, week.

Compared with healthy volunteers, patients with UC displayed increased systemic inflammatory biomarkers (SAA and IL-6), neutrophil activation biomarkers (NGAL, OSM, and IL-8), and Th17 (IL-17A and IL-22), Th2 (IL-5), and Th1 (IFN-γ) cytokines (Figure 2B; statistical tests for associations were not performed).

Patients with UC also had higher gene-expression-derived plasmablast and cell proliferation factors, and lower gene-expression-derived B cell, pDC, and NK-cell factors than healthy volunteers.

#### UC biologic-experienced versus biologic-naive patients

Biologic-experienced patients had a more severe inflammatory phenotype across most biomarkers at baseline than the biologic-naive patients. Specifically, they had increased systemic inflammation biomarkers (SAA, CRP, IL-6, and platelet cell counts), neutrophil activation biomarkers (all measured except for IL-8), and Th17 cytokines (IL-17A and IL-23) (Figure 2B).

There were also significant differences in factors that mediate anti-inflammatory effects, with IL-2 and IL-10 levels elevated, and TGF-β1 levels decreased, in biologic-experienced versus biologic-naive patients.

Gene-expression-derived B cell, cell proliferation, T-cell activation, and pDC factors were lower for biologic-experienced than biologic-naive patients, while immunoglobulin (Ig)M levels were higher.

#### Pharmacodynamic effects of concomitant drugs at baseline

At baseline, patients taking corticosteroids displayed lower levels of Th17 (IL-17A and IL-22) and Th2 (IL-5 and eosinophil counts) cytokines than those not taking corticosteroids (Figure 2C). However, higher levels of neutrophil activation markers (serum OSM, neutrophil counts, and serum and fecal calprotectin), SAA, and IL-10 were observed in patients taking corticosteroids compared with matched patients who were not.

In addition, patients taking corticosteroids displayed higher levels of the gene-expression-derived effector B-cell factor and lower levels of B cell, T-cell activation, pDC, and NK-cell factors compared with matched patients who were not.

The only circulatory biomarkers associated with immunomodulator use were monocyte counts, NGAL, and serum calprotectin. B-cell and NK-cell factors were also significantly lower in patients using immunomodulators versus those who were not.

### Effects of Filgotinib Treatment on Biomarkers

#### Changes in protein biomarkers

Filgotinib significantly lowered levels of almost all circulating biomarkers associated with UC pathobiology (bringing them closer to levels observed in healthy volunteers) at weeks 4 and 10 relative to placebo (Figure 2D and Table S2), especially at the 200 mg dose. Most importantly, there was a consistent decrease in all systemic inflammatory biomarkers (except gp130), neutrophil activation biomarkers, Th17- and Th2-related factors, and IgM levels. These effects were similar in biologic-naive and biologic-experienced patients. Of note, filgotinib treatment did not significantly reduce any cytokines associated with anti-inflammatory activity (IL-2, IL-10, and TGF-β).

Fecal calprotectin and lactoferrin levels were also significantly decreased with filgotinib treatment relative to placebo at week 10 (week 4 samples were unavailable).

The anti-inflammatory effect of filgotinib on humoral immunity was demonstrated via the systemic decrease in levels of IgM, plasmablasts, and cell proliferation factor at the expense of an increase in B-cell and effector B-cell factors (Figure 2D and Figure S1A).

Furthermore, a decrease in NK-cell and T-cell activation factors and an increase in pDC factor were observed at the filgotinib 200 mg dose (Figure 2D and Figure S1A).

#### Changes in protein biomarkers in clinical responders

**Figure 3.**
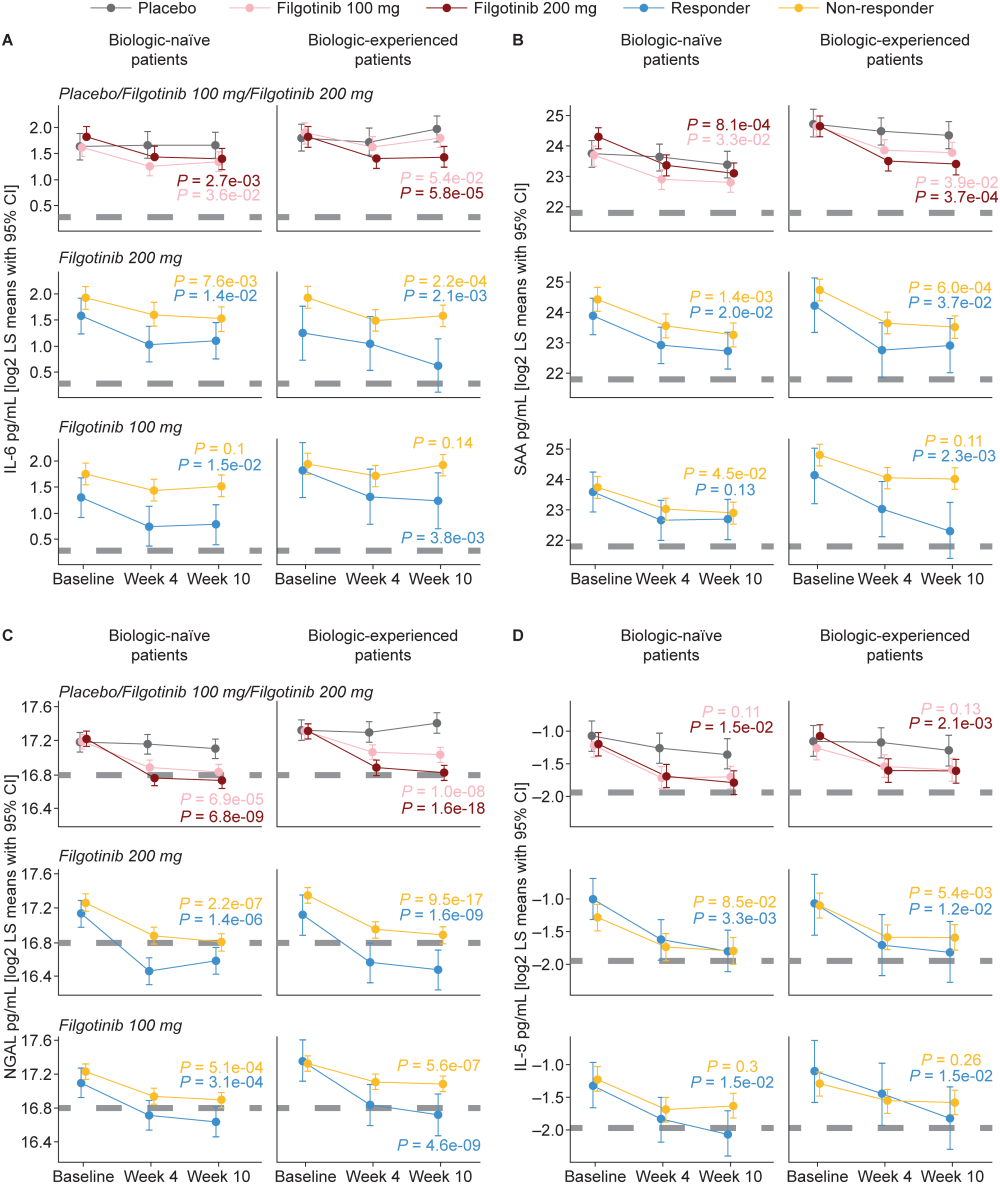
Filgotinib treatment effect in biologic-naive and biologic-experienced patients on serum protein biomarkers (A) IL-6, (B) SAA, (C) NGAL, and (D) IL-5. Gray dashes indicate estimated biomarker levels for healthy volunteers. *P* values reflect filgotinib treatment changes from baseline to week 10 compared with placebo. Abbreviations: CI, confidence interval; IL, interleukin; LS, least-squares; NGAL, neutrophil gelatinase-associated lipocalin; SAA, serum amyloid A.

We plotted individual biomarker levels in patients receiving filgotinib (200 mg or 100 mg) or placebo at baseline and weeks 4 and 10 (Figure 3) to better understand what happens to these biomarkers in clinical responders versus nonresponders.

Using the systemic biomarker IL-6 as an example, filgotinib 200 mg significantly reduced IL-6 levels at week 10 compared with placebo in both biologic-naive and biologic-experienced patients, especially in responders, where levels approached values detected in healthy volunteer samples (Figure 3A). Even nonresponders had trends toward reduced IL-6 levels with filgotinib treatment, indicating some disease benefits at the molecular level. There were also trends toward reduced IL-6 levels in patients receiving filgotinib 100 mg, although these were less often statistically significant compared with filgotinib 200 mg. Trends similar to those for IL-6 were also observed for SAA, NGAL, and IL-5 (Figure 3B–D).

### Effects of Baseline Biomarker Levels on Filgotinib Treatment Response

**Figure 4.**
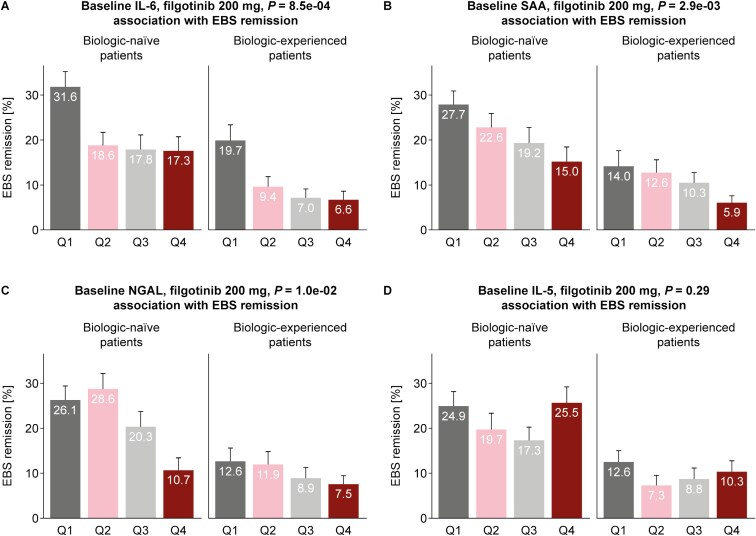
Baseline biomarker values associated with EBS remission at week 10 in patients receiving filgotinib 200 mg ([A] IL-6, [B] SAA, [C] NGAL, and [D] IL-5). Panels indicate EBS remission rates per biomarker concentration quartile (Q1 [25% with lowest levels]–Q4 [25% with highest levels]). Error bars indicate the standard error of the mean. *P* values reflect associations between baseline biomarker levels and EBS remission at week 10. Abbreviations: EBS, endoscopy/bleeding/stool; IL, interleukin; NGAL, neutrophil gelatinase-associated lipocalin; Q, quarter; SAA, serum amyloid A.

To gain insight into which patients are more likely to respond to filgotinib 200 mg, we examined EBS remission rates at week 10 in relation to biomarker concentrations by quartile in patients receiving filgotinib 200 mg (Figure 4). Higher baseline concentrations of IL-6, SAA, and NGAL were associated with lower EBS remission rates with similar trends in both biologic-naive and biologic-experienced patients. In contrast, IL-5 level did not display any baseline association with EBS remission (Figure 4D).

**Figure 5.**
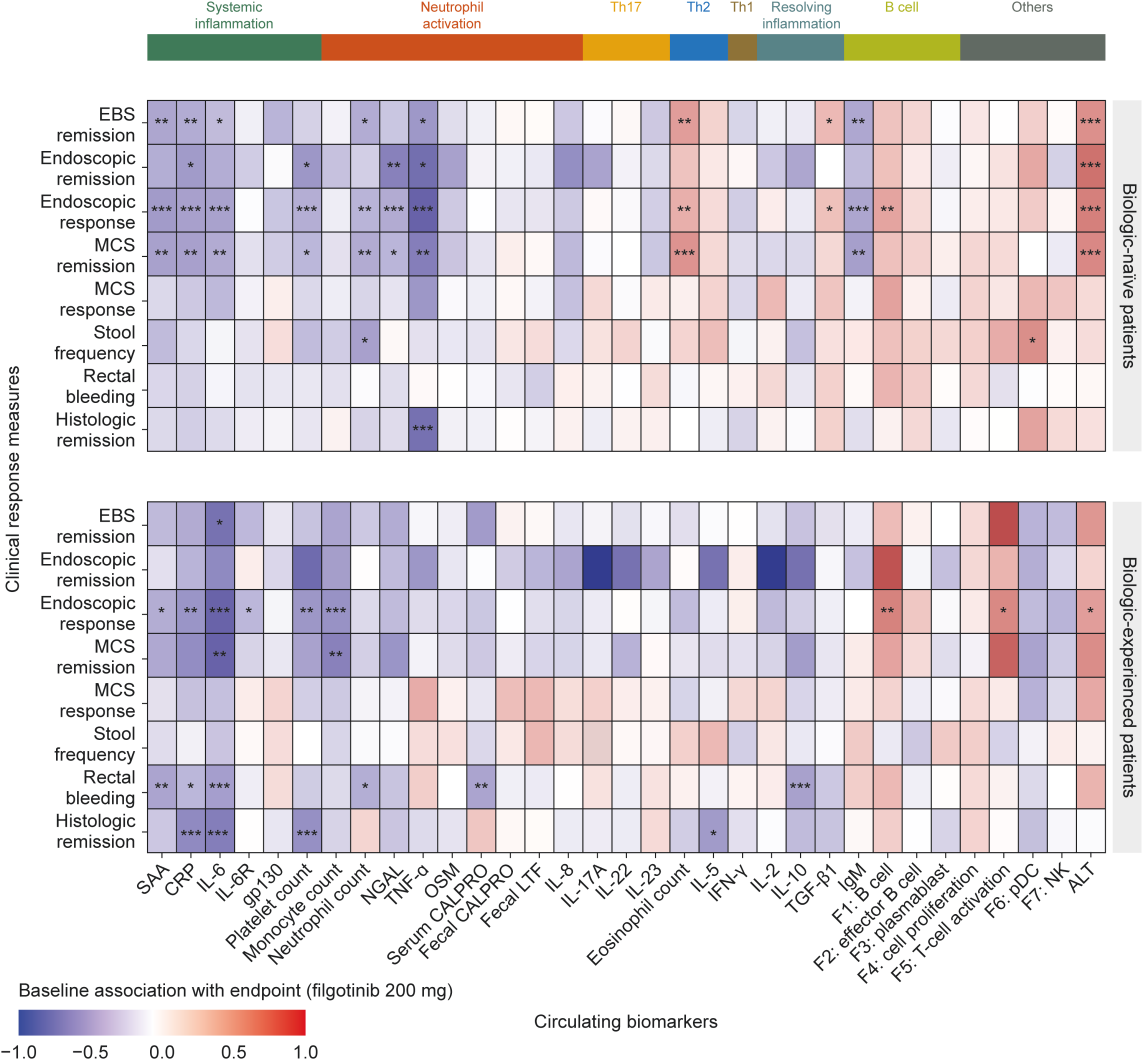
Association between the baseline serum biomarker levels and clinical response/outcomes at week 10 after filgotinib 200 mg treatment. *FDR < 0.2, **FDR < 0.1, and ***FDR < 0.05. Abbreviations: ALT, alanine transaminase; CALPRO, calprotectin; CRP, C-reactive protein; EBS, endoscopy/bleeding/stool; F, factor; FDR, false discovery rate; gp, glycoprotein; IFN, interferon; Ig, immunoglobulin; IL, interleukin; IL-6R, IL-6 receptor; LTF, lactoferrin; MCS, Mayo Clinic Score; NGAL, neutrophil gelatinase-associated lipocalin; NK, natural killer; OSM, oncostatin M; pDC, plasmacytoid dendritic cell; SAA, serum amyloid A; TGF, transforming growth factor; Th, T helper; TNF, tumor necrosis factor.

In addition, we evaluated the association between baseline biomarker levels and all clinical endpoints at week 10 (Figure 5). Lower baseline levels of several systemic inflammatory biomarkers (CRP, IL-6, and platelet counts) were positively associated with week 10 endoscopic response in both biologic-experienced and biologic-naive patients, while lower baseline levels of the systemic inflammatory biomarker SAA and neutrophil activation biomarkers (NGAL, TNF-α, and neutrophil counts) were positively associated with week 10 endoscopic response in biologic-naive patients only. Higher levels of all these biomarkers were associated with more severe UC at baseline (Figure 1). Conversely, higher baseline levels of the B-cell factor were positively associated with week 10 endoscopic response in both cohorts (Figure 5), while lower baseline levels were associated with higher UC severity (Figure 1). Thus, overall, more severe UC was less clinically responsive to filgotinib 200 mg.

In biologic-naive patients, higher baseline TGF-β1 levels were positively associated with more severe UC at baseline, and there was a trend toward an association between higher baseline TGF-β1 levels and week 10 endoscopic response in patients receiving filgotinib 200 mg (Figures 1 and 5). In addition, lower baseline IgM levels were associated with week 10 endoscopic response in biologic-naive patients, despite no associations with UC severity at baseline.

### Mediation Analysis of Serum Protein Data

**Figure 6.**
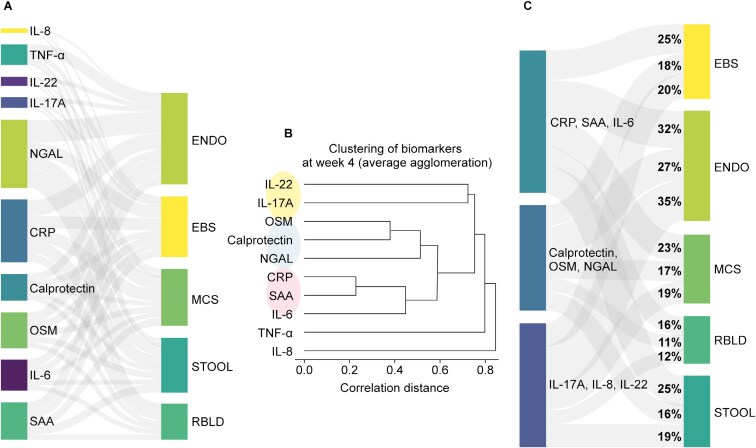
Causal modeling between early biomarker changes following filgotinib treatment. (A) Week 4 serum biomarkers that are associated with clinical disease improvements at week 10, (B) clustering of serum biomarkers at week 4 using correlation distance, and (C) associations between biomarkers grouped by the EFA at week 4 and subsequent clinical disease improvements at week 10. Connector width indicates the proportion of total effect associated with the “indirect” path (ie, via corresponding biomarkers at week 4). Statistical associations with FDR < 0.1 are shown. Abbreviations: CRP, C-reactive protein; EBS, endoscopy/bleeding/stool; ENDO, endoscopic subscore; FDR, false discovery rate; Filgo, filgotinib; IL, interleukin; LS, least-squares; MCS, Mayo Clinic Score; NGAL, neutrophil gelatinase-associated lipocalin; OSM, oncostatin M; RBLD, rectal bleeding subscore; SAA, serum amyloid A; STOOL, stool frequency subscore; TNF, tumor necrosis factor.

Lower week 4 levels of systemic inflammatory biomarkers (SAA, CRP, IL-6, and platelet counts) and neutrophil activation biomarkers (NGAL, TNF-α, and neutrophil counts) were positively associated with subsequent clinical response at week 10 (Figure S3). We performed mediation analyses to assess whether filgotinib treatment produced UC clinical improvements at week 10 by virtue of its effects on certain biomarkers at week 4 (Figure 6). The effects of filgotinib 200 mg on 10 serum protein biomarkers at week 4 were identified as potential mediators of improved clinical disease activity at week 10 (Figure 6A). Reduced levels of SAA, CRP, IL-6, NGAL, and OSM at week 4 were the main contributors to the subsequent associated improvements at week 10 from baseline in all 5 clinical scores (EBS, MCS, and endoscopic, rectal bleeding, and stool frequency subscores). IL-8, TNF-α, IL-22, and IL-17A were associated with endoscopic and stool frequency subscores but not with rectal bleeding subscores.

The causal effects identified were not independent; they represent several clusters (Figure 6B). Therefore, we identified 3 latent factors using the EFA. All 3 latent factors of serum protein biomarkers were associated with week 10 clinical improvement (Figure 6C). These comprised systemic inflammatory biomarkers (highest loading for CRP, SAA, and IL-6), neutrophil-associated biomarkers (highest loading for calprotectin, OSM, and NGAL), and Th17 cytokines (highest loading for IL-17A and IL-22). Causal modeling thus suggests that inhibition of these 3 molecular pathways at week 4 leads to subsequent improvements in UC clinical scores at week 10.

## Discussion

This preplanned analysis of the phase 2b/3 SELECTION trial revealed that UC pathobiology is characterized by increased levels of biomarkers for systemic inflammation, neutrophil activation, Th17, Th2, and Th1 pathway activation, and mediators of resolved inflammation. Importantly, these findings from serum samples collectively mirror inflammation and UC manifestations in disease tissues of the colon.^3–5,21,22^ Filgotinib significantly impacted many circulating biomarkers related to UC pathobiology. Most importantly, mediation analysis revealed that early (week 4) changes in a subset of these biomarkers, related to systemic inflammation, neutrophil activation, and the Th17 cytokine pathway, may be key mediators of week 10 clinical improvements associated with filgotinib treatment. These findings support the possibility of using circulating biomarkers as a proxy for invasive mucosal assessments in patients with UC, and for potentially identifying patients who are more likely to respond to treatment with filgotinib.

Filgotinib reduced the levels of nearly all studied proinflammatory biomarkers (some to near “normal” levels) associated with UC pathobiology at baseline, especially at the 200 mg dose, which impacted biomarker levels to a greater extent than the 100 mg dose in our analysis (Supplementary Methods, Results and Figure S4), and has shown the greatest efficacy in clinical trials.^16^ In addition, mediation analysis identified week 4 reductions in systemic inflammation (CRP, SAA, and IL-1), neutrophil activation (calprotectin, OSM, and NGAL), and Th17 cytokines (IL-17A and IL-22) as potential early mediators of clinical response at week 10. Interestingly, filgotinib did not significantly reduce levels of cytokines associated with anti-inflammatory activity (IL-10 and TGFβ-1). Thus, JAK1 inhibition may reduce UC severity by simultaneously reducing systemic inflammation, Th17 cytokines (also observed with ustekinumab),^23^ and neutrophil infiltration, while maintaining some anti-inflammatory pathways. Importantly, although elevated Th1 and Th2 cytokines are known hallmarks of inflammation in UC (confirmed in this study), mediation analysis suggests that they may play a “nondriver” role in the clinical effects of filgotinib.

At the whole-blood gene-expression level, lower B cell, effector B cell, pDC, and NK-cell factors, as well as higher plasmablast and cell proliferation factors, were also found to be a feature of UC. Filgotinib increased B cell, effector B cell, and pDC factors at the expense of reduced plasmablast, cell proliferation, T-cell activation, and NK-cell factors. Altered NK-cell number, function, and phenotype have also been observed with JAK inhibitors.^24–26^ Moreover, increased B-cell numbers in peripheral blood have been observed with filgotinib treatment in patients with rheumatoid arthritis,^27^ and filgotinib suppresses human B-cell differentiation in vitro.^28^ B-cell responses have been shown to be highly dysregulated in patients with UC, with one study reporting an expansion of naive B cells and IgG^+^ plasma cells in the mucosal B-cell compartment in patients with UC compared with healthy controls.^29^ Fewer pDCs in blood and more gut recruitment of pDCs have been observed in patients with active versus inactive inflammatory bowel disease (IBD) and compared with healthy controls.^30^ In addition, patients in remission in this study had circulating pDC proportions similar to those observed in control participants.^30^ Although the exact role of infiltrating pDCs in IBD remains unclear,^31^ Arimura et al. have postulated their involvement in the induction of colonic inflammation that promotes the accumulation of inflammatory phagocytes.^32^

In terms of signaling pathways, our hallmark pathway analysis showed early reductions in gene signatures for IL-6-JAK-STAT3 signaling with filgotinib 200 mg relative to placebo in both biologic-naive and biologic-experienced patients (Supplementary Results and Figure S5). Consistent with this, the concentration of the proinflammatory cytokine IL-6 was significantly reduced in biologic-naive and biologic-experienced patients following filgotinib 200 mg treatment compared with placebo. In support of our findings, Shien et al. demonstrated that filgotinib suppresses STAT3 activation.^33^ STAT3 suppression has also been shown to inhibit IL-6 production.^34^ In addition, our hallmark pathway analysis indicated increased Wnt/β-catenin and NOTCH signaling with filgotinib 200 mg relative to placebo at week 10 in both biologic-naive and biologic-experienced patients. These findings are consistent with the mechanism of action of filgotinib, with JAK 1/2 previously shown to regulate the Wnt signaling pathway genes.^35^ Wnt and/or NOTCH signaling pathways are crucial for the maintenance of intestinal stem cells, secretory cells, and epithelial tight junctions.^36^ Dysregulation of these signaling pathways in patients with IBD contributes to impaired intestinal healing.^36^

Overall, biologic-experienced patients with UC displayed a more severe "molecular" disease profile than biologic-naive patients, with elevated systemic inflammation and neutrophil infiltration biomarkers (particularly TNF-α) and Th17 cytokines. These data are consistent with studies showing that non-responders to anti-TNF therapy have a more severe inflammatory profile than responders, and with clinical observations that treatment-refractory patients tend to have a worse clinical disease course and higher rates of surgery.^37^ Interestingly, higher baseline levels of TGF-β1, an anti-inflammatory mediator,^38^ were observed in biologic-naive rather than in biologic-experienced patients, and there was a trend toward better clinical response to filgotinib 200 mg at week 10 in biologic-naive patients who had higher baseline TGF-β1 levels. Thus, higher TGF-β1 levels in biologic-naive patients may contribute to a less severe inflammatory phenotype versus biologic-experienced patients and a greater potential for achieving clinical response to filgotinib. TGF-β1 was also positively associated with disease severity. Although this may initially seem counterintuitive, increased TGF-β1 has been observed previously in patients with UC and may reflect greater stimulation of anti-inflammatory processes in the presence of more severe inflammation.^38^

Another finding of interest in our study relates to T-cell activation. As expected, filgotinib 200 mg significantly reduced the T-cell activation profile. However, baseline T-cell activation was negatively correlated with disease severity and was reduced in biologic-experienced versus biologic-naïve patients with UC. These results may reflect heterogenous activities of different types of T cells, or factors such as T-cell exhaustion and senescence, with reductions or increases in the T-cell activation gene-expression profile not necessarily being a simple indicator of “more” or “less” severe disease. Indeed, our data strongly suggest differing patterns of perturbed inflammatory pathways in these patients reflecting substantial immune diversity in UC, which contrasts with a much simpler “one-dimensional” view, where each immune pathway is more significantly perturbed in biologic-experienced patients than in biologic-naïve patients.

Corticosteroid use at baseline was associated with reduced eosinophil counts and reduced levels of Th17 and Th2 cytokines and TNF-α, as well as reduced pDC gene-expression factors, while immunomodulator use was associated with reduced serum calprotectin and NGAL levels and monocyte counts. The different effects of these treatments on circulating biomarkers are not entirely surprising, given that these drugs reduce inflammation via different mechanisms. Immunomodulators (in this case, methotrexate, azathioprine, and 6-mercaptopurine) inhibit the replication of rapidly dividing cells, including immune cells, by interfering with their DNA synthesis. Corticosteroids, on the other hand, bind to glucocorticoid receptors, enabling the nuclear localization of activated glucocorticoid receptor–steroid complexes that cause transcriptional repression and activation of numerous genes and interfere with nuclear factor-κB and activator protein-1 signaling, 2 important inflammatory transcriptional regulators (reviewed by Coutinho et al).^39^ In contrast to filgotinib, both immunomodulators and corticosteroids left many key markers of UC pathobiology unaffected. Furthermore, levels of SAA, fecal and serum calprotectin, and IL-10 were elevated with corticosteroid use in the current analysis, and neutrophil cell counts were increased, which may reflect processes contributing to steroid resistance or side effects of steroid use. SAA is a clinical biomarker for systemic inflammation.^40^ Corticosteroids are known to suppress inflammation, but prolonged treatment can induce SAA production via Toll-like receptor signaling.^41,42^ Important limitations of our analysis by corticosteroid use are that samples were collected at baseline after a prolonged period of use (vs just 4 and 10 weeks of filgotinib treatment), and that inadequate clinical response, loss of response to, or intolerance of corticosteroids or immunosuppressants were selection criteria for entry into the SELECTION trial.^16^

There have been conflicting reports regarding serum protein biomarkers in UC to date, likely reflecting small sample sizes and variations in patient characteristics such as disease severity.^43,44^ Similarly, although other studies have assessed the impacts of advanced therapies (eg, infliximab, vedolizumab, etrasimod, and ustekinumab) on circulating biomarkers, the sample sizes have been relatively small, and the range of biomarkers assessed has been limited.^45–48^ Key strengths of the current study thus include the large sample size available for analysis and the well-characterized nature of the population. However, because only patients with moderate to severe UC were eligible for the SELECTION trial, correlations between UC pathobiology and clinical disease activity metrics were limited to active disease. Furthermore, the present study does not assess the potential acute effects of filgotinib treatment at earlier time points (such as weeks 1 and 2), which have been described in patients with Crohn’s disease.^49^

In the present study, filgotinib impacted many circulating biomarkers related to pathological features of UC. Importantly, biomarkers related to systemic inflammation, neutrophil activation, and the Th17 cytokine pathway appear to be early mediators of clinical improvement with filgotinib treatment and may be worthy of future exploration as potential predictors of filgotinib treatment response.

## Supplementary Data

Supplementary data is available at *Inflammatory Bowel Diseases* online.

izae278_suppl_Supplementary_Material

## Data Availability

Gilead Sciences shares anonymized individual patient data upon request or as required by law or regulation with qualified external researchers based on submitted curriculum vitae and reflecting nonconflict of interest. The request proposal must also include a statistician. Approval of such requests is at Gilead Science’s discretion and is dependent on the nature of the request, the merit of the research proposed, the availability of the data, and the intended use of the data. Data requests should be sent to datarequest@gilead.com.
