# Supplementary material for: Mediators of Filgotinib Treatment Effects in Ulcerative Colitis: Exploring Circulating Biomarkers in the Phase 2b/3 SELECTION Study
Source: Inflamm Bowel Dis. 2024 Dec 4;31(4):1095–108. doi: 10.1093/ibd/izae278 (PMC11985404; doi:10.1093/ibd/izae278)

**Supplementary Data**

# Supplementary Methods

## Biomarker collection and assessments

### Whole-blood RNA sequencing biomarkers

RNA was isolated from whole blood using the PAXgene 96 Blood RNA Kit (Qiagen, Hilden, Germany). Globin mRNA was removed using the GLOBINClear-Human Kit (ThermoFisher, Waltham, MA, USA). RNA sequencing (RNA-seq) libraries were prepared using the TruSeq Stranded mRNA Library Prep kit (Illumina, San Diego, CA, USA) and sequenced on the Illumina HiSeq 2500 platform. Raw read sequence (FASTQ) files were mapped to Genome Reference Consortium Human Build 38 and quantitated using Salmon. Quality control metrics evaluating RNA samples, raw sequences, and alignments were generated by FastQC, Spliced Transcripts Alignment to a Reference (STAR), Salmon, Sequence Alignment/Map (SAM) tools, Qualimap 2, and in-house R scripts.

Overall, 16 097 genes were selected for further analysis based on observing ≥10 mRNA reads for each gene in ≥1% of total samples.

## Statistical comparisons and correlations

### Correlation of baseline biomarkers with ulcerative colitis disease activity

Biomarker levels, including for serum and fecal proteins, whole-blood cell counts, and seven factors identified from the exploratory factor analysis (EFA) of RNA-seq data, were correlated with baseline clinical disease activity and histology measures to identify associations with ulcerative colitis disease severity. Clinical disease activity measures comprised endoscopy/bleeding/stool (EBS) score, Mayo Clinic Score (MCS), partial MCS, and Mayo Clinic subscores (endoscopic, rectal bleeding, and stool frequency). Histology scores comprised the Geboes Score and its components (structural changes, chronic inflammatory infiltrate, lamina propria eosinophils and neutrophils, neutrophils in the epithelium, crypt destruction, and erosions or ulcerations), and the Robarts Histopathology Index.

Correlations between baseline biomarker levels and clinical disease activity measures were calculated using Spearman’s rank correlation and displayed as a heatmap, with the false discovery rate (FDR) to illustrate the strength of correlations.

### Between-group comparisons of baseline biomarkers

A linear mixed-effects model was applied to compare biomarker levels between biologic‑naïve and biologic-experienced patients at baseline adjusted for covariates such as age, sex, baseline endoscopic score, duration of disease, US versus non-US region, smoking status, and use of concomitant medicines.

A linear mixed-effects model was also used to assess the effects of corticosteroids or immunomodulators on baseline biomarker levels. Because patients were not stratified according to corticosteroid or immunomodulator use in SELECTION, propensity score matching was performed to account for differences in baseline covariates, including age, sex, race, cohort, region, biologic status, smoking status, duration of disease, endoscopic score (for corticosteroid use analysis), and Robarts Histopathology Index (for immunomodulator use analysis).

Results were displayed as a heatmap with the FDR to illustrate the strength of associations.

### Effects of filgotinib treatment on biomarkers and single-sample gene set enrichment analysis scores, and assessment of dose responses

Linear mixed-effects models were used to identify effects of filgotinib treatment on biomarker levels and single-sample gene set enrichment analysis (ssGSEA) scores calculated using whole-blood RNA-seq data, as described above. A treatment effect was defined as a biomarker change from baseline to week 10 or week 4 in the filgotinib (FIL) 200 mg or 100 mg treatment arms compared with placebo (PBO), as follows:

$$\left( FIL_{Week10 or Week4}-FIL_{Baseline} \right)-\left( PBO_{Week10 or Week 4}-PBO_{Baseline} \right)$$

Results were displayed as a heatmap with the FDR to illustrate the strength of associations. Corresponding results plots were displayed as log2-transformed least-squares means with 95% confidence intervals and the *P* value.

The slope of the fitted linear regression line was calculated to quantitatively compare filgotinib 100 mg and 200 mg doses, as well as the effect of filgotinib on circulating biomarkers at week 4 versus week 10, and in biologic-naïve versus biologic-experienced patients. Corresponding scatter plots were displayed with 95% confidence intervals.

### Effects of biomarker levels on filgotinib treatment response

Associations between biomarker levels at baseline or at week 4 and clinical response at week 10 following filgotinib 200 mg treatment were explored using a logistic regression model. The covariates used in the model included age, sex, race, smoking status, and concomitant medication use. Clinical responders were defined as those who achieved EBS remission (endoscopic subscore of 0 or 1, rectal bleeding subscore of 0, and a ≥1-point decrease in stool frequency from baseline to achieve a subscore of 0 or 1) at week 10.

Results were displayed as a heatmap with the FDR to illustrate the strength of associations.

A logistic regression model was employed to evaluate the association between baseline biomarker levels and EBS remission at week 10 in patients who received filgotinib 200 mg. Biologic-naïve and biologic-experienced patients were stratified by quartile values of biomarker levels at baseline.

# Supplementary Results

## Effects of filgotinib treatment on biomarkers

### Assessment of dose responses

Placebo-adjusted treatment effects were compared between filgotinib 100 mg and 200 mg doses in order to detect dose responses (**Supplementary Figure 4A** and **B**). Filgotinib 100 mg impacted biomarker levels to a lesser extent than filgotinib 200 mg in both biologic-naïve (slope 0.66) and biologic-experienced (slope: 0.64) patients.

When comparing placebo-adjusted treatment effects at week 10 versus week 4, both the 200 mg and 100 mg doses displayed slopes closer to 1 in both biologic-naïve and biologic-experienced patients. Thus, filgotinib had exerted most of its effects on circulating biomarkers by week 4 (**Supplementary Figure 4C–F**).

In addition, the treatment effect of filgotinib on circulating biomarkers appeared to be similar in both biologic-naïve and biologic-experienced patients (**Supplementary Figure 4G** and **H**).

### Changes in gene signatures identified by hallmark pathway analysis

Filgotinib treatment effects relative to placebo were analyzed for changes in hallmark pathway activity scores, indicating changes in gene expression signatures (**Supplementary Figure 5**). As expected, most immune-related pathway activity scores, including IL-6-JAK-STAT3 and inflammatory response signaling pathways, decreased relative to placebo following treatment with filgotinib 200 mg in both biologic-naïve and biologic-experienced patients.

The decrease in cell-cycle related pathway activity scores, as well as G2M checkpoint and E2F targets in biologic-experienced patients (**Supplementary Figure 5**), mirror the profile of cell proliferation factors identified by the EFA (**Supplementary Figure 1A**).

Of note, filgotinib 200 mg increased Wnt/β-catenin and NOTCH signaling activity scores relative to placebo at week 10 in both biologic-naïve and biologic-experienced patients. Moreover, at weeks 4 and 10, TGF-β signaling increased and cell proliferation (cell cycle-related gene expression) decreased in biologic-experienced patients.

# Supplementary Tables and Figure Legends

**Supplementary Table 1.** Summary of serum biomarkers, vendors, and the fraction of samples within the limits of quantification.

| Biomarker | Full name | Vendor | Method | Proportion within LOQ range (all samples) | Proportion within LOQ range (baseline only) |
| --- | --- | --- | --- | --- | --- |
| CALPRO | Calprotectin (ng/mL) | Nexelis | ELISA | 99.49% | 99.42% |
| CRP | C-reactive protein (mg/dL) | Labcorp | ELISA | 93.12% | 96.99% |
| gp130 | Glycoprotein 130 (pg/mL) | Quanterix | Simoa Planar Array | 99.97% | 100.00% |
| IFN-ɣ | Interferon gamma (pg/mL) | Quanterix | Simoa Planar Array | 81.22% | 84.42% |
| IL-1B | Interleukin-1B (pg/mL) | Quanterix | Simoa Planar Array | 46.81% | 51.74% |
| IL-17A | Interleukin-17A 1-Plex Array (pg/mL) | Quanterix | Simoa Planar Array | 99.01% | 99.49% |
| IL-2 | Interleukin-2 1-Plex Array (pg/mL) | Quanterix | Simoa Planar Array | 90.46% | 89.33% |
| IL-4 | Interleukin-4 (pg/mL) | Quanterix | Simoa Planar Array | 35.49% | 36.58% |
| IL-10 | Interleukin-10 (pg/mL) | Quanterix | Simoa Planar Array | 99.94% | 99.83% |
| IL-12p70 | Interleukin-12p70 (pg/mL) | Quanterix | Simoa Planar Array | 46.33% | 49.45% |
| IL-22 | Interleukin-22 (pg/mL) | Quanterix | Simoa Planar Array | 100.00% | 100.00% |
| IL-23 | Interleukin-23 (pg/mL) | Quanterix | Simoa Planar Array | 83.21% | 83.15% |
| IL-5 | Interleukin-5 (pg/mL) | Quanterix | Simoa Planar Array | 60.04% | 70.53% |
| IL-6 | Interleukin-6 (pg/mL) | Quanterix | Simoa Planar Array | 99.80% | 99.66% |
| IL-6R | Interleukin-6 receptor (pg/mL) | Quanterix | Simoa Planar Array | 100.00% | 100.00% |
| IL-8 | Interleukin-8 (pg/mL) | Quanterix | Simoa Planar Array | 100.00% | 100.00% |
| NGAL | Neutrophil gelatinase-associated lipocalin (pg/mL) | Quanterix | Simoa Planar Array | 100.00% | 100.00% |
| OSM | Oncostatin M (pg/mL) | Nexelis | ELISA | 94.90% | 96.04% |
| SAA | Serum amyloid A-1 protein (pg/mL) | Quanterix | Simoa Planar Array | 99.69% | 99.66% |
| TGF-β1 | Transforming growth factor β1 (pg/mL) | Quanterix | Simoa Planar Array | 100.00% | 100.00% |
| TNF-α | Tumor necrosis factor α (pg/mL) | Quanterix | Simoa Planar Array | 99.97% | 100.00% |

Serum and fecal biomarkers that had >50% of observations outside the limits of quantification were excluded from the statistical analysis.

Abbreviations: ELISA, enzyme-linked immunosorbent assay; LOQ, limit of quantification.

**Supplementary Table 2.** Summary of serum and fecal biomarkers with significantly^a^ different concentrations based on association with disease severity and with filgotinib treatment versus placebo.

|  |  | **Biologic-naïve patients with UC** | | | | **Biologic-experienced patients with UC** | | | |
| --- | --- | --- | --- | --- | --- | --- | --- | --- | --- |
|  | **Association with disease severity^b^** | **Filgotinib 100 mg vs placebo** | | **Filgotinib 200 mg vs placebo** | | **Filgotinib 100 mg vs placebo** | | **Filgotinib 200 mg vs placebo** | |
| **Week** | Baseline | 4 | 10 | 4 | 10 | 4 | 10 | 4 | 10 |
| **Systemic inflammation** | CRP  SAA  IL-6  Platelet count  gp130 | **CRP**  **SAA**  **IL-6**  **Platelet count** | **CRP**  **SAA**  **IL-6** | **CRP**  **SAA**  **IL-6**  **Platelet count**  **IL-6R** | **CRP**  **SAA**  **IL-6**  **gp130** | **CRP**  **SAA**  **Platelet count** | **CRP**  **SAA**  **IL-6**  **Platelet count** | **CRP**  **SAA**  **IL-6**  **Platelet count**  **IL-6R** | **CRP**  **SAA**  **IL-6**  **Platelet count**  **IL-6R** |
| **Neutrophil activation** | NGAL  TNF-α  OSM  Serum CALPRO  Fecal CALPRO  Fecal LTF  Monocyte count  Neutrophil count | **NGAL**  **TNF-α**  N/A  N/A  **Neutrophil count** | **NGAL**  **TNF-α**  **Fecal CALPRO**  **Fecal LTF**  **Neutrophil count** | **NGAL**  **TNF-α**  **OSM**  **Serum CALPRO**  N/A  N/A  **Neutrophil count**  **IL-8** | **NGAL**  **TNF-α**  **OSM**  **Serum CALPRO**  **Fecal CALPRO**  **Fecal LTF**  **Monocyte count** | **NGAL**  **TNF-α**  OSM  Serum  CALPRO  N/A  N/A | **NGAL**  **TNF-α**  **Fecal CALPRO**  **Fecal LTF**  **Monocyte count** | **NGAL**  **TNF-α**  **OSM**  **Serum CALPRO**  N/A  N/A  **Monocyte count** | **NGAL**  **TNF-α**  **OSM**  **Serum CALPRO**  **Fecal CALPRO**  **Fecal LTF**  **Monocyte count** |
| **Th17** | IL-17A  IL-22 | **IL-17A**  **IL-23**  **IL-22** | **IL-17A**  **IL-23** | **IL-17A**  **IL-23**  **IL-22** | **IL-17A**  **IL-23** |  | **IL-17A**  **IL-23** | **IL-17A**  **IL-23** | **IL-17A**  **IL-23**  **IL-22** |
| **Th2** | IL-5 | **IL-5**  **Eosinophil count** | **Eosinophil count** | **IL-5**  **Eosinophil count** | **IL-5**  **Eosinophil count** | **IL-5**  **Eosinophil count** | **Eosinophil count** | **IL-5**  **Eosinophil count** | **IL-5**  **Eosinophil count** |
| **Th1/IFN-γ** | IFN-γ |  |  |  |  | **IFN-γ** |  |  |  |
| **Resolving inflammation** | TGF-β1  IL-2  IL-10 |  | **TGF-β1** | **IL-2** |  |  |  | **IL-2** |  |
| **B** |  | **IgM** | **IgM** | **IgM** | **IgM** | **IgM** | **IgM** | **IgM** | **IgM** |
| **Others** | ALT |  |  |  | **ALT** |  |  | **ALT** | **ALT** |

Red text indicates relative increases in biomarker concentrations. Black text indicates relative decreases in biomarker concentrations.

^a^Bold text indicates FDR <0.1 and normal text indicates FDR >0.1 and *P* <0.05 for the significance of concentration changes following filgotinib treatment.

^b^Association with disease severity was demonstrated by a significant baseline correlation (FDR <0.1) with either MCS or PMCS.

Abbreviations: ALT, alanine transaminase; CALPRO, calprotectin; CRP, C-reactive protein; FDR, false discovery rate; gp, glycoprotein; IFN, interferon; Ig, immunoglobulin; IL, interleukin; IL-6R, IL-6 receptor; LTF, lactoferrin; MCS, Mayo Clinic Score; N/A, not available; NGAL, neutrophil gelatinase‑associated lipocalin; OSM, oncostatin M; PMCS, partial MCS; SAA, serum amyloid A; TGF, transforming growth factor; Th, T helper; TNF, tumor necrosis factor; UC, ulcerative colitis.

**Supplementary Figure 1.** Treatment effects of filgotinib on selected factors with A, a clear biological interpretation identified from whole-blood RNA sequencing, and B, biological interpretation of gene-expression derived factors. Loadings of gene-expression derived factors were applied to the internal PBMC cell atlas to reveal relative cell specificity. The signal for the highest matching cell was normalized at 1. *FDR <1 × 10^−1^, **FDR <1 × 10^−3^, and ***FDR <1 × 10^−6^. Abbreviations: aMBc, atypical memory B cells; BnCS, class-switched naïve B cells; BnUS, unswitched naïve B cells; cMBc, class-switched classical memory B cells; coDC, conventional dendritic cells; CI, confidence interval; F, factor; FDR, false discovery rate; FIL, filgotinib; gadT, gammadelta T cells; Ig, immunoglobulin; LS, least-squares; Mbim, IgM^+^ IgM^–^ classical memory B cells; MoCl, classical monocytes; MoIn, intermediate monocytes; MoNC, non-classical monocytes; NK, natural killer; NKhi, CD56hi NK cells; NKlo, CD56low NK cells; NS, not significant; pBcs, class-switched plasmablasts; PBMC, peripheral blood mononuclear cell; pDC, plasmacytoid dendritic cell; T4cm, central memory CD4^+^ T cells; T4em, effector memory CD4^+^ T cells; T4nv, naïve CD4^+^ T cells; T4ra, CD45RA^+^ effector memory CD4^+^ T cells; T8cm, central memory CD8^+^ T cells; T8em, effector memory CD8^+^ T cells; T8nv, naïve CD8^+^ T cells; T8ra, CD45RA^+^ effector memory CD8^+^ T cells; traB, transitional B cells; Treg, T regulatory cells.


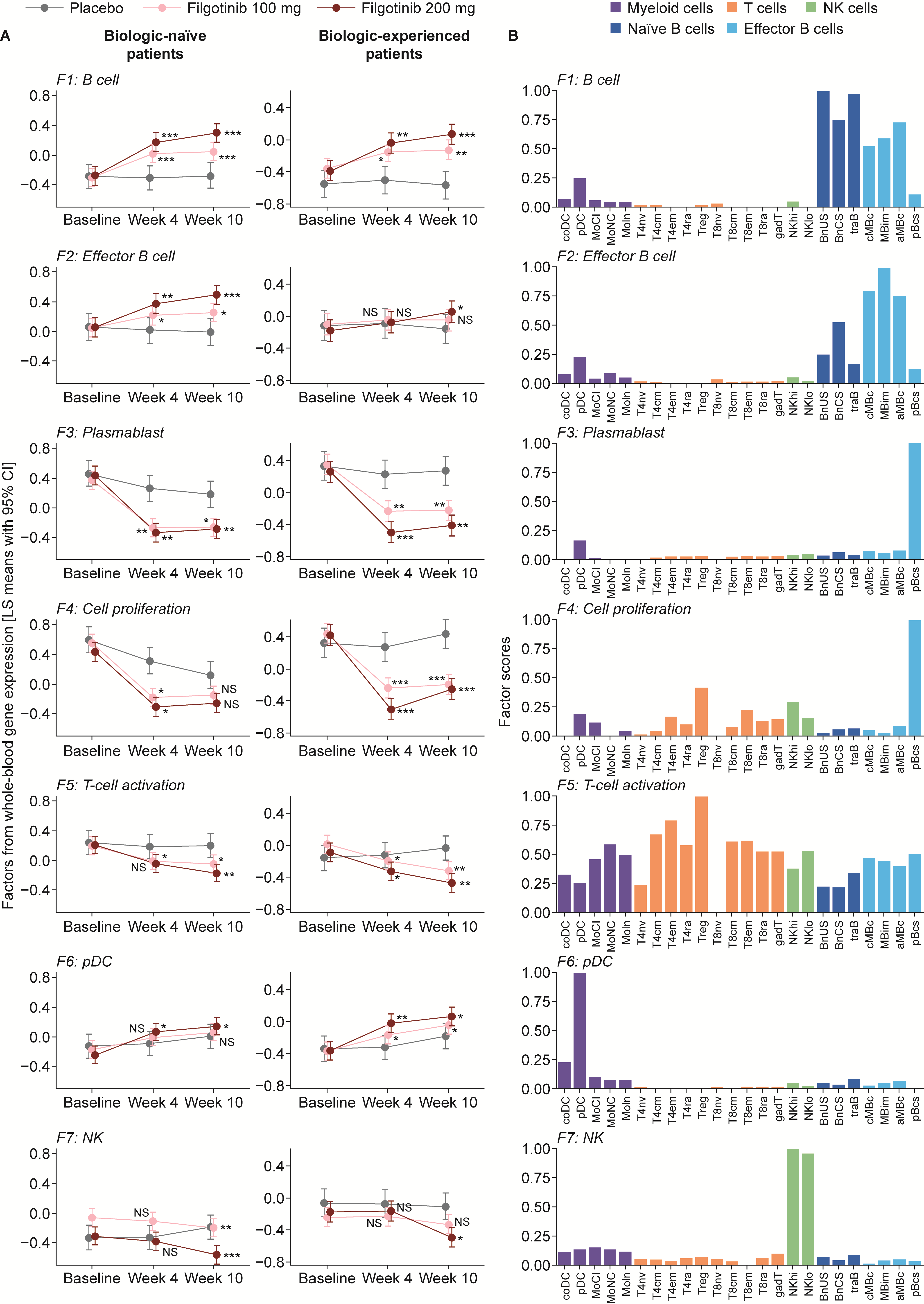


**Supplementary Figure 2.** Causal modelling framework.


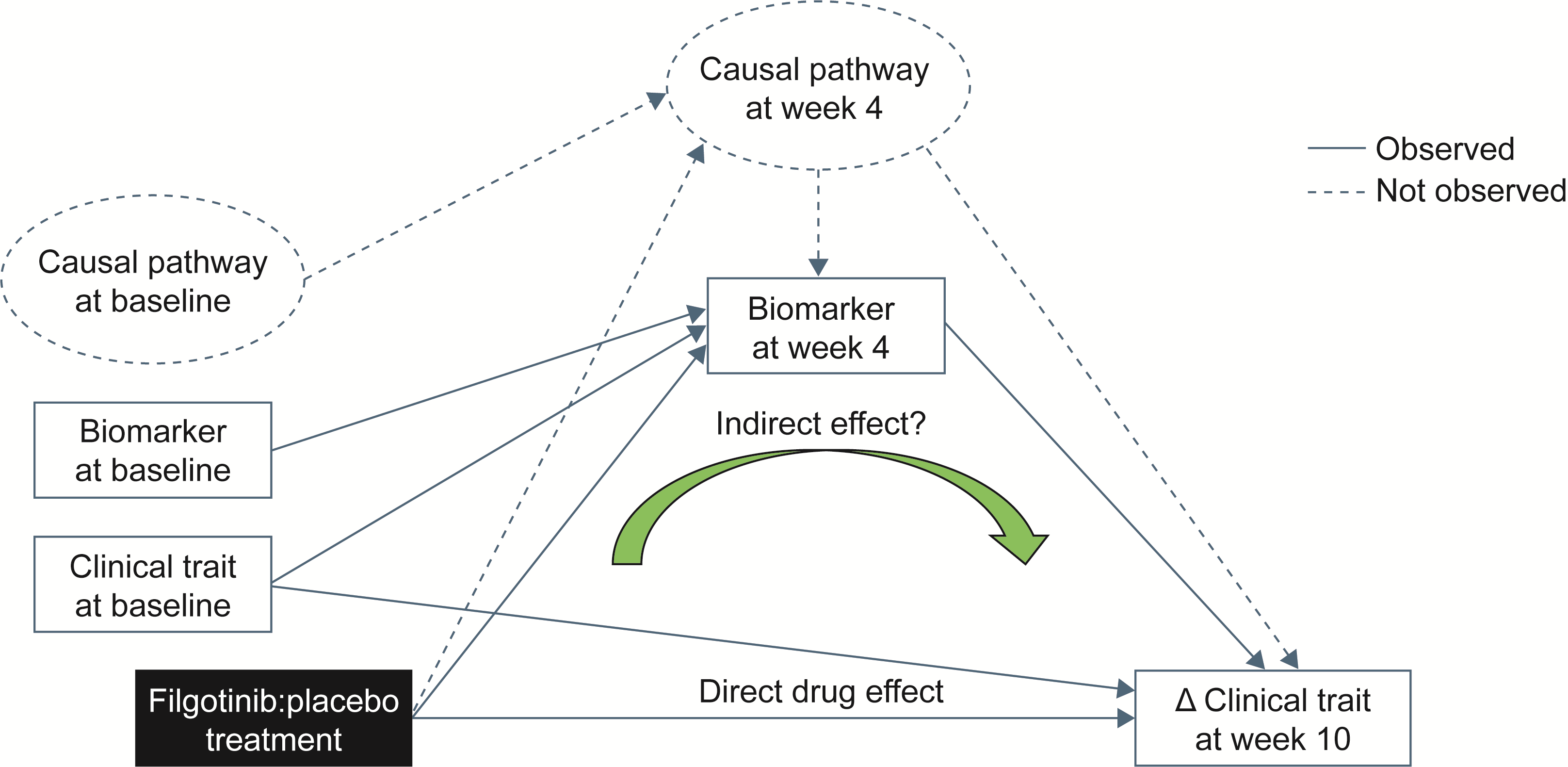


**Supplementary Figure 3.** Association between the week 4 serum biomarker levels and clinical response at week 10 after filgotinib 200 mg treatment. *FDR <0.05, **FDR <1 × 10^−3^, and ***FDR <1 × 10^−6^. Abbreviations: ALT, alanine transaminase; CALPRO, calprotectin; CRP, C-reactive protein; EBS, endoscopy/bleeding/stool; F, factor; FDR, false discovery rate; gp, glycoprotein; IFN, interferon; Ig, immunoglobulin; IL, interleukin; IL-6R, IL-6 receptor; LTF, lactoferrin; MCS, Mayo Clinic Score; NGAL, neutrophil gelatinase-associated lipocalin; NK, natural killer; OSM, oncostatin M; pDC, plasmacytoid dendritic cell; SAA, serum amyloid A; TGF, transforming growth factor; TNF, tumor necrosis factor.


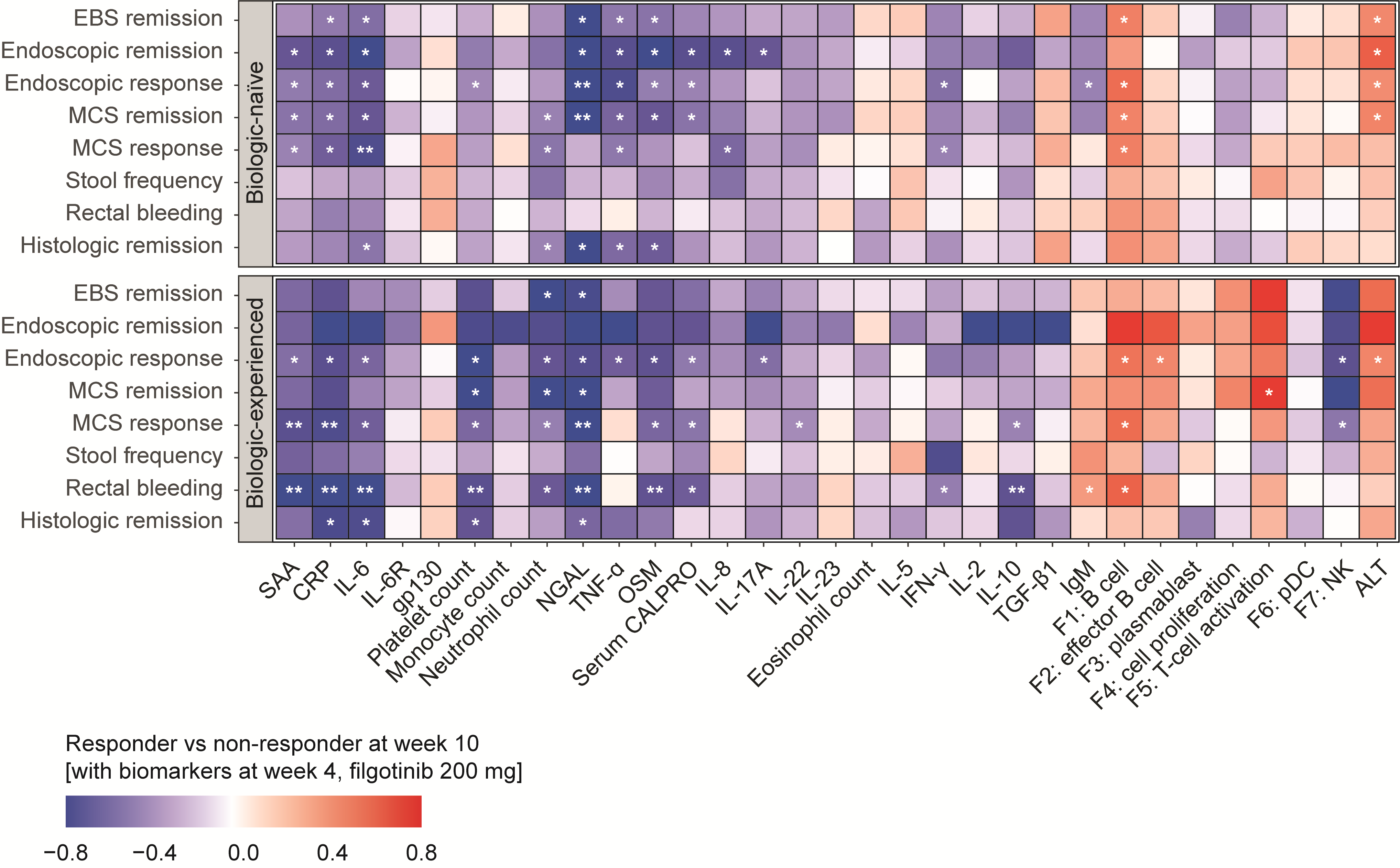


**Supplementary Figure 4.** Comparisons of placebo-adjusted filgotinib treatment effects across all biomarkers. Filgotinib 100 mg versus 200 mg at week 10 in A, biologic-naïve patients and B, biologic-experienced patients. Week 10 versus week 4 in biologic-naïve patients following C, filgotinib 100 mg and D, filgotinib 200 mg. Week 10 versus week 4 in biologic-experienced patients following E, filgotinib 100 mg and F, filgotinib 200 mg. Biologic-experienced versus biologic-naïve patients at week 10 following G, filgotinib 200 mg and H, filgotinib 100 mg. Abbreviations: CALPRO, calprotectin; CI, confidence interval; CRP, C‑reactive protein; F, factor; IL, interleukin; LTF, lactoferrin; NGAL, neutrophil gelatinase-associated lipocalin; OSM, oncostatin M; SAA, serum amyloid A.


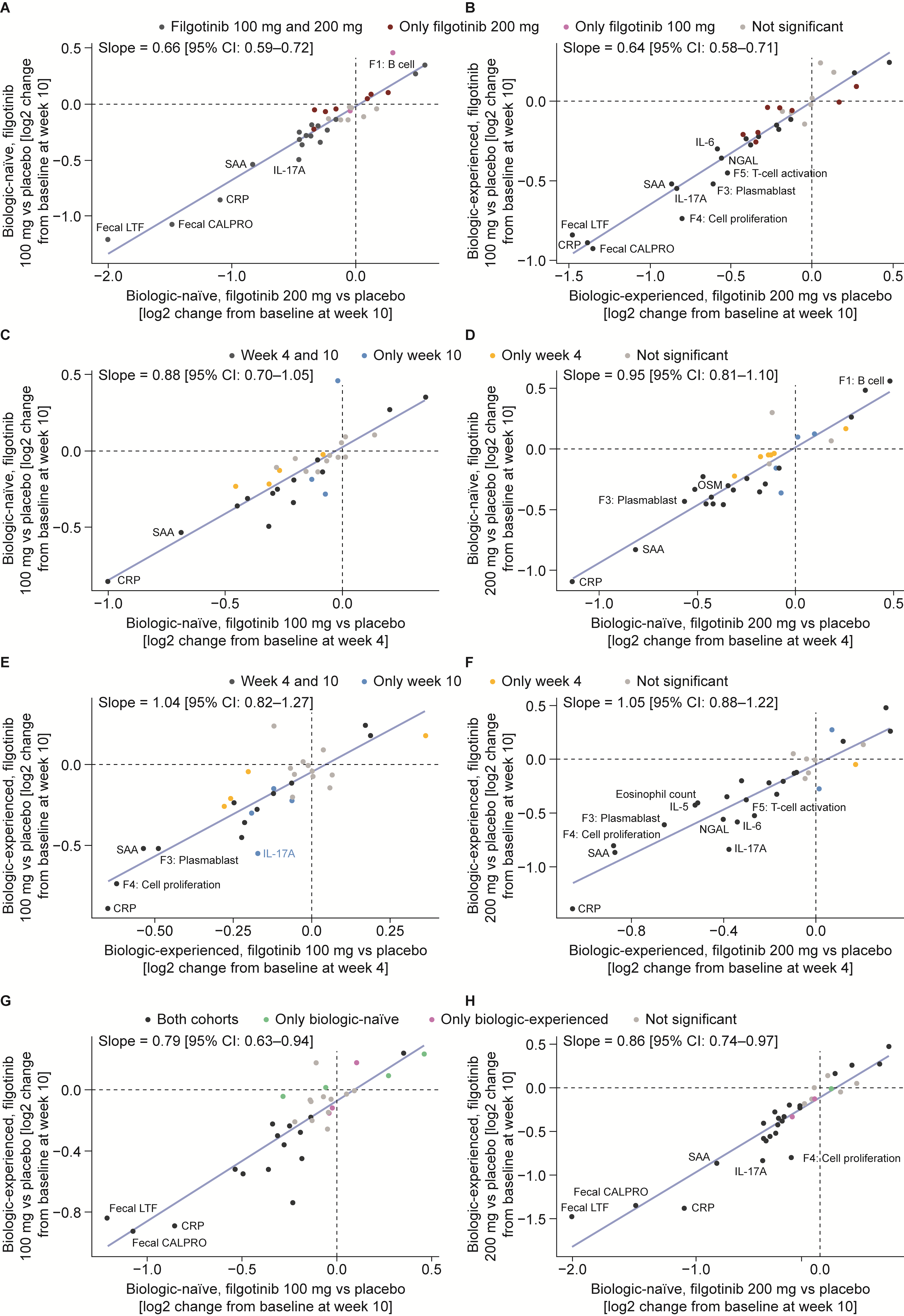


**Supplementary Figure 5.** Placebo-adjusted treatment effects of filgotinib 200 mg at weeks 4 and 10 on hallmark pathways derived from whole-blood RNA-seq data. Pathways were collected from MSigDB hallmark gene sets. Significance indicates FDR <0.05. Abbreviations: dn, down; FDR, false discovery rate; IL, interleukin; JAK, Janus kinase; KRAS; Kirsten rat sarcoma virus; MSigDB, molecular signature database; mTOR, mechanistic target of rapamycin; MTORC1, mechanistic target of rapamycin complex 1; NFKB, nuclear factor kappa B; PIK3, phosphoinositide 3 kinase; RNA-seq, RNA sequencing; ssGSEA, single-sample gene set enrichment analysis; STAT, signal transducer and activation of transcription; TGF, transforming growth factor; TNF, tumor necrosis factor; UV, ultraviolet.


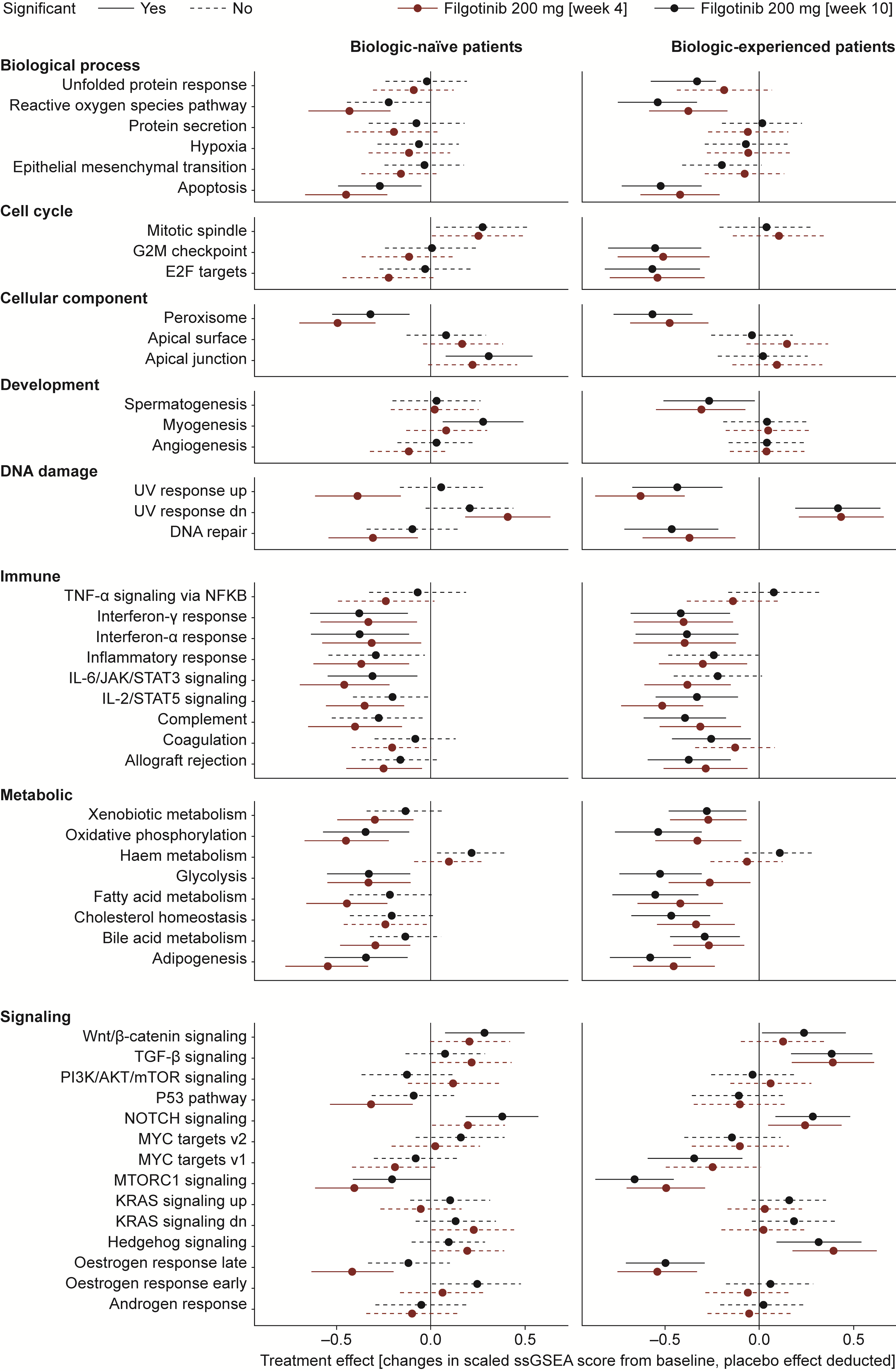

Supplement: izae278_suppl_Supplementary_Material [file izae278_suppl_supplementary_material.docx]
